# A cost-effective, community-based, mosquito-trapping scheme that captures spatial and temporal heterogeneities of malaria transmission in rural Zambia

**DOI:** 10.1186/1475-2875-13-225

**Published:** 2014-06-07

**Authors:** Chadwick H Sikaala, Dingani Chinula, Javan Chanda, Busiku Hamainza, Mulenga Mwenda, Isabel Mukali, Mulakwa Kamuliwo, Neil F Lobo, Aklilu Seyoum, Gerry F Killeen

**Affiliations:** 1 National Malaria Control Centre, Chainama Hospital College Grounds, Off Great East road, P.O. Box 32509 Lusaka, Zambia; 2 Vector Biology Department, Liverpool School of Tropical Medicine, Pembroke Place, Liverpool L3 5QA, UK; 3 Department of Biological Sciences, University of Notre Dame, Eck Institute for Global Health, Notre Dame, IN 46556, USA; 4 Ifakara Health Institute, Environmental Health and Ecological Sciences Thematic Group, PO Box 53, Ifakara, Morogoro, United Republic of Tanzania

**Keywords:** Community-based, Trapping scheme, Cost-effective, *Anopheles funestus*

## Abstract

**Background:**

Monitoring mosquito population dynamics is essential to guide selection and evaluation of malaria vector control interventions but is typically implemented by mobile, centrally-managed teams who can only visit a limited number of locations frequently enough to capture longitudinal trends. Community-based (CB) mosquito trapping schemes for parallel, continuous monitoring of multiple locations are therefore required that are practical, affordable, effective, and reliable.

**Methods:**

A CB surveillance scheme, with a monthly sampling and reporting cycle for capturing malaria vectors, using Centers for Disease Control and Prevention light traps (LT) and Ifakara Tent Traps (ITT), were conducted by trained community health workers (CHW) in 14 clusters of households immediately surrounding health facilities in rural south-east Zambia. At the end of the study, a controlled quality assurance (QA) survey was conducted by a centrally supervised expert team using human landing catch (HLC), LT and ITT to evaluate accuracy of the CB trapping data. Active surveillance of malaria parasite infection rates amongst humans was conducted by CHWs in the same clusters to determine the epidemiological relevance of these CB entomological surveys.

**Results:**

CB-LT and CB-ITT exhibited relative sampling efficiencies of 50 and 7%_,_ respectively, compared with QA surveys using the same traps. However, cost per sampling night was lowest for CB-LT ($13.6), followed closely by CB-ITT ($18.0), both of which were far less expensive than any QA survey (HLC: $138, LT: $289, ITT: $269). Cost per specimen of *Anopheles funestus* captured was lowest for CB-LT ($5.3), followed by potentially hazardous QA-HLC ($10.5) and then CB-ITT ($28.0), all of which were far more cost-effective than QA-LT ($141) and QA-ITT ($168). Time-trends of malaria diagnostic positivity (DP) followed those of *An. funestus* density with a one-month lag and the wide range of mean DP across clusters was closely associated with mean densities of *An. funestus* caught by CB-LT (P < 0.001).

**Conclusions:**

CB trapping schemes appear to be far more affordable, epidemiologically relevant and cost-effective than centrally supervised trapping schemes and may well be applicable to enhance intervention trials and even enable routine programmatic monitoring of vector population dynamics on unprecedented national scales.

## Background

**Figure 1:**
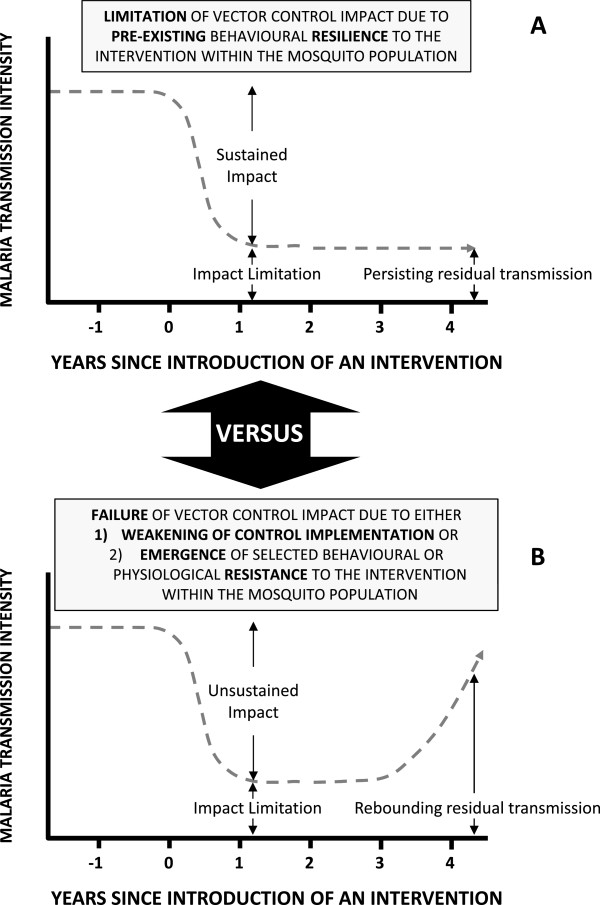
**A schematic illustration of the differing trajectories of impact of an intervention upon malaria transmission by a vector population under the distinctive scenarios of either (A) stable limitation of sustained impact arising from expression of pre-existing behavioural traits within a resilient vector population, or (B) failure of impact and resurgence of malaria transmission when, either intervention programme implementation quality and coverage weakens, or selected behavioural or physiological traits emerge within an increasingly resistant, rebounding vector population**[2,4,6,7][12]**.**

Despite the impressive successes of long-lasting insecticidal nets (LLINs) and indoor residual spraying (IRS), which selectively target malaria vectors when they feed or rest inside human habitations, these front line vector control tools have rarely achieved complete elimination of malaria outside of areas that had marginal transmission levels to begin with [1-3]. These fundamental limits of what can be achieved with IRS or LLINs are primarily defined by the behavioural traits of mosquitoes [2,4-10], most of which appear to have always been present in these populations [2,4-7] so they are better described as pre-existing behavioural *resilience* (Figure 1A) [6,7]. On the other hand, recent modelling analyses [11] have illustrated how apparently altered distributions of feeding times and locations following scale-up of LLIN or IRS cannot be simply explained in terms of deferred feeding by hungry mosquitoes and may represent emergence of selected, heritable behavioural *resistance* in the strict sense (Figure 1B) [6,7]. Furthermore, resurgent malaria has been repeatedly associated with, not only failures of implementation and funding for vector control programmes, but also with emergence of physiological resistance to insecticides [12]. It is therefore crucial to distinguish between such fundamental *limitations* of a given vector control strategy, reflecting incomplete but nevertheless valuable levels of *sustainable* impact (Figure 1A), and a genuine *failure* of an intervention programme that results in *rebounding* vector populations and malaria transmission (Figure 1B).

The only way in which suppression or resurgence of malaria transmission can be unambiguously attributed to the success or failure of interventions to control responsible vectors will be to monitor their population dynamics longitudinally. Currently, across sub-Sahara Africa, almost all monitoring of vector populations is limited to detecting physiological resistance to prioritize optimal selection of active ingredients for intra-domiciliary insecticidal-based interventions. It has therefore been suggested that robust longitudinal sentinel surveillance systems need to be established so that national malaria control programmes (NMCPs) can continually monitor physiological and behavioural traits, and assess their relevance to intervention selection, by evaluating their impact upon the population dynamics of target vector species [6,13].

However, the cost of implementing adult mosquito surveillance through conventional teams of specialist entomologists may be prohibitive in impoverished African countries [14,15]. Conventional longitudinal entomological monitoring strategies rely operationally upon trained specialist technical staff managed centrally usually by academic or research institutions, so they are usually limited in both their geographic scope and the frequency of sampling at any survey location. The availability and cost of the expert human resources required to sustain such specialist teams is also limiting [14-16]. Mosquito species composition, abundance and transmission potential is not only altered by successful implementation of vector control measures [2,6,8,13], it also varies dramatically geographically and seasonally. It is therefore difficult to envision how conventional, centralized entomological surveillance teams could capture such spatial and temporal patterns in a representative manner on national scales because they simply cannot reach all sentinel survey locations often enough to provide a robust representation of longitudinal trends at each one.

Decentralized systems that adapt cost-effective trapping methods to local, longitudinal application by resident community-based (CB) staff therefore represent an attractive alternative [14,15]. Implementation of CB trapping schemes presents two important challenges: 1) selection of traps, and protocols for their use, that are safe practical and convenient enough for CB staff to apply them reliably in the absence of daily supervision, and 2) independent quality assurance (QA) of this unsupervised surveillance process so that the accuracy and limitations of the derived data can be quantified as a prerequisite to critical interpretation. To date, however, only one CB mosquito-trapping scheme, designed to support a municipal-scale, larval source management programme in Dar es Salaam in Tanzania, has been critically evaluated through both QA of the derived entomological data and appraisal of its epidemiological relevance in terms of its ability to predict malaria infection risk among humans [15]. This first validated CB trapping scheme was also more sensitive, in terms of total numbers of mosquito caught, than the centrally supervised scheme used to conduct QA, because it was much more intensive and at the same time spatially extensive [15]. Furthermore, CB trapping results in Dar es Salaam were predictive of malaria risk infection amongst humans despite the fact that vector populations were remarkably sparse in this low transmission urban area [15]. However, the generalizability of this study to a wider variety of settings is not only limited by its local geographic scope, but also by the fact that it relied on entirely upon a locally designed Ifakara Tent Trap (ITT) [17,18] because this was shown to be the only safe, sufficiently sensitive capture method in this context where *Anopheles gambiae* is the predominant species maintaining transmission [19].

Over the last decade, Zambia has made substantial progress toward implementing an ambitious strategic plan aiming to protect every at-risk individual in the country against malaria with either LLINs or IRS [20]. As insecticide resistance has now been clearly identified within the country, it is essential to develop a sustainable platform to monitor vector species composition, behaviour and transmission capacity on a national scale for the first time. Recent comparative evaluations of various mosquito-trapping methods, in rural south-east Zambia [21], where malaria transmission is primarily maintained by *Anopheles funestus*, demonstrated that the Centers for Disease Control and Prevention miniature Light Trap (LT) and ITT [18] both performed reasonably well as methods for capturing host-seeking mosquitoes and also suggested that they could be applied across a much larger geographic area through a more practical and scalable CB system. This manuscript describes an evaluation of the applicability of CB trapping schemes, using these two candidate capture methods, to assess their effectiveness for sampling malaria vectors across different times and locations, as well as their overall cost effectiveness and ability to predict human malaria infection risk in the same rural Zambian transmission system [21].

## Methods

### Study area

**Figure 2:**
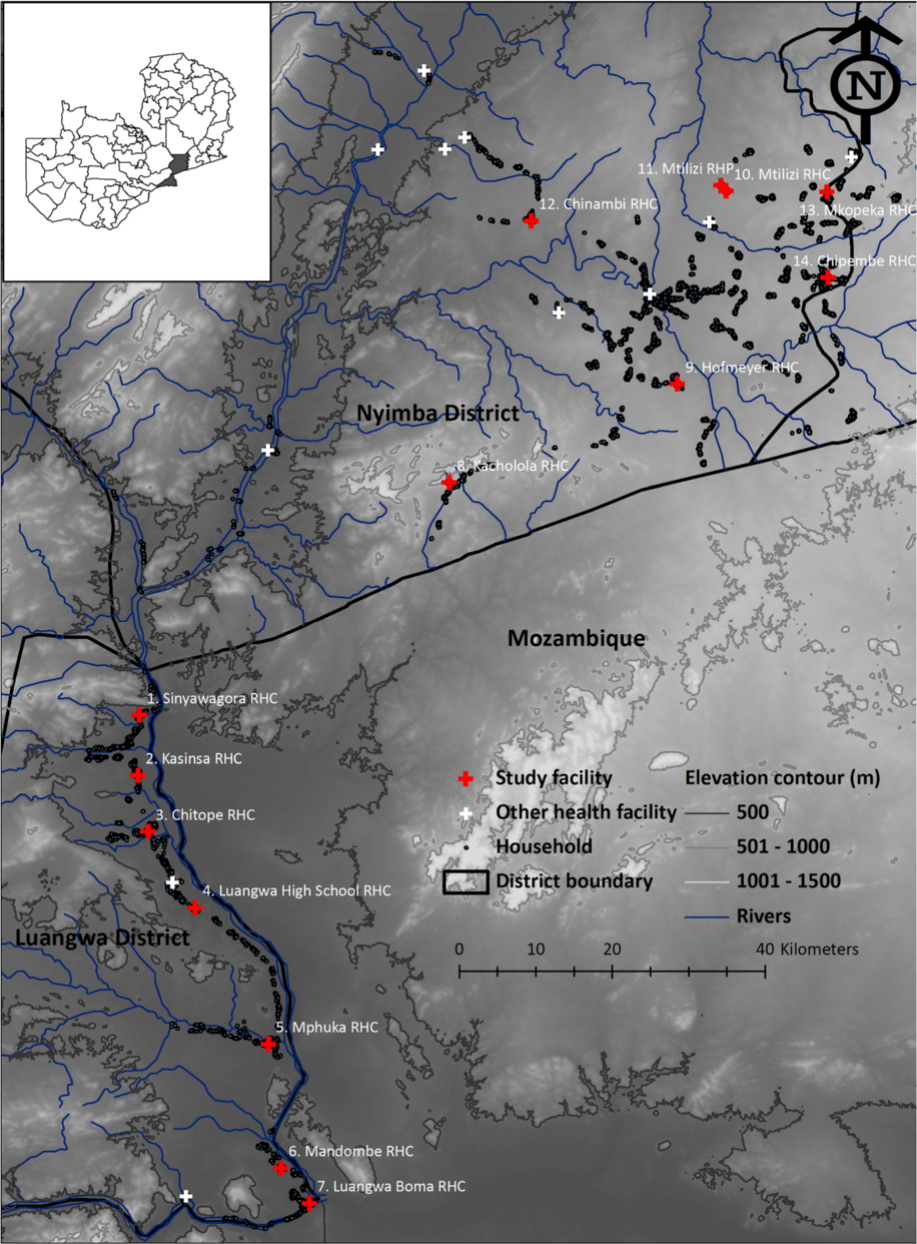
Location of study site, and numbered survey clusters around health facilities, in Zambia.

The study was conducted in Luangwa and Nyimba districts, located approximately 255 km and 325 km, respectively, east of Lusaka, the capital city of Zambia (Figure 2). There are about 25,000 and 85,000 inhabitants in Luangwa and Nyimba, respectively, who predominantly practice seasonal farming, fishing and animal husbandry as their primary livelihood [22]. Malaria prevalence in this part of Zambia ranges from 9 to 22%, with by far the lowest prevalence in the flat, sandy southern half of Luangwa [23]. Within this study area, intensive monitoring of malaria infection among the humans, and of human-biting mosquito densities in and around their houses, was carried out on a monthly survey cycle by resident community health workers (CHWs) in 14 population clusters centred around health facilities between January 2011 and March 2013 [22].

Between 2005 and 2012, Luangwa and Nyimba districts received repeated mass distributions of LLINs, complemented by routine distribution through antenatal clinics. As a result, 66 and 43%, respectively of children under five years of age in Luangwa and Nyimba reported using a net the previous night by 2010 [24]. IRS was implemented between October and November 2010, using deltamethrin (K-Othrine WG® 250, Bayer Environmental Science, South Africa) in the south of Luangwa district. At the same time of year in 2011, some of these villages in southern Luangwa district were sprayed with lambdacyhalothrin (Icon® 10 Capsule Suspension (CS) formulation, Syngenta Crop Protection AG, Switzerland) while others, as well as several in Nyimba district, were sprayed with an emulsifiable concentrate (EC) formulation of the organophosphate pirimiphos-methyl (Actellic® EC, Syngenta Crop Protection AG, Switzerland) to mitigate against the resistance to pyrethroids among *An. funestus* populations in the area [25]. A year later 2012, the same regime was applied except that the selection of villages sprayed with pirimiphos methyl in Nymiba was changed and, in some of them, the EC formulation was replaced with a micro-encapsulated formulation of the same active ingredient (Actellic® 300CS, Syngenta Crop Protection AG, South Africa).

### Longitudinal malaria parasite surveillance in the human population

Fourteen population clusters of approximately 1,000 residents were selected in both districts (seven per district), each centred around a public sector health facility, in which each household was visited monthly by a CHW offering testing and treatment for malaria [22]. Three CHWs were recruited for this task, of which two enrolled approximately 60 households while the third, who was also responsible for CB mosquito trapping as described below, enrolled 45 households for parasitological surveillance. CHWs enlisted households in order of their proximity to the health facility and collected small finger-stick blood samples from all consenting and assenting household members who were present on a designated date each month for each household and tested on the spot using the MAL Pf® Rapid Diagnostic Test (RDT) kit (ICT Diagnostic, Cape Town, South Africa) that detects histidine rich protein-2 (HRP-2) antigen. All individuals that tested positive in the field for the presence of malaria parasite antigen were provided with artemether-lumefantrine (Coartem®, Norvatis Pharma AG, Basel, Switzerland) free-of-charge in accordance with the national guidelines. Between these active visits, individuals who felt sick or had symptoms were encouraged to seek medical care from their assigned CHW or the nearest health facility so cases were also detected passively. All the recruited CHWs were remunerated as casual labourers at a rate of ZMW 350 ($66.9) per month. Mean diagnostic positivity rates of residents in individual clusters tested during monthly activity visits to their households ranged from 6 to 47% with an overall mean of 20.3% (11,851/58,500) across all age groups [22], approximately consistent with the recent National Malaria Indicator Survey [23] that describes a mean infection prevalence of 19.5% in cross sectional household surveys in the rural districts of Zambia.

### Community-based mosquito-trapping scheme

In order to assess the effectiveness of CB surveillance of adult mosquito populations, one of the three CHWs in each cluster (specifically the ones with 45 households to survey) had additional training in basic entomology. One exception was at Luangwa High School (cluster 4), where two out of the three CHWs were engaged in conducting entomological surveillance of adult mosquito populations with one covering 45 while the other 60 households in the surveys of infection among the human residents. Fifteen houses per cluster for mosquito trapping were selected semi-arbitrarily to be well distributed across the cluster, with the exception of Luangwa High School where this figure was doubled to 30 due to the involvement of an additional CHW in mosquito trapping. Therefore, the targeted number of trapping nights per house per month was one. The cluster, village, and household codes, and household owner name for each household were recorded for all 299 households where CB surveys of mosquitoes were conducted. A consistent date of the month for mosquito trapping using the LT and ITT at each house was pre-agreed with each household head. The LTs were placed inside the house on the foot end of an occupied sleeping space already covered with LLIN at a height of approximately 1.5 m above the floor whilst an adult male from the same household occupied an ITT placed immediately outside, approximately 5 m away from the house where the LT was installed. The only occasion volunteers where replaced was in cases of illness, resignation or unreliability. Due to the inconvenience of the bulkiness of the ITT [15,21] CHWs were provided with spare parts to maintain their bicycles to facilitate transport of the traps from one household to another during the study period. Mosquito traps were set up in the evenings and captured mosquitoes were collected by aspiration as early as was convenient the next morning. CHWs were trained to sort mosquitoes to genus level by eye, to store them over silica, and to keep it desiccated. *Anopheles* specimens were stored individually in 1.5 ml microcentrifuge tubes while culicines were pooled in ziplock bags. Based on this crude morphological classification, the numbers of mosquitoes caught were recorded on a simple form by the CHW.

A team from the centralized National Malaria Control Centre (NMCC) entomological team collected the mosquito samples from all of the clusters once per month and delivered them to the central laboratory at the NMCC in Lusaka. At the central laboratory, anopheline mosquito samples were subjected to further morphological identification [26] and the data entered into an Excel sheet. Then the *An. gambiae* complex and *An. funestus* group were taken to the molecular laboratory for further analysis and long-term storage. CB mosquito trapping was conducted continuously from January 2011 to April 2013 in Luangwa and from April 2011 to April 2013 in Nyimba district.

### Quality assurance surveys of the community-based trapping

In order to assess the validity of the CB trapping schemes using the LTs and ITTs, a QA team was assembled towards the end of the study. This team was recruited selectively from among the most experienced CHWs who were involved during the previous trap efficacy study in Chisobe village of Luangwa district. None of these team members had any other responsibilities within this particular study and were supervised by a technical team of trained entomologists from the central level at NMCC.

To validate the CB trapping schemes, the QA team visited the same households that the CB team had placed their traps a day or two earlier. The trapping efficacy of LT and ITT applied by the QA team, and their efficiency and effectiveness as applied by the CHWs, were compared with the gold standard human landing catches technique (HLC) [27] conducted by one male adult volunteer indoors and another outdoors. As described above, every month, on a date that was pre-agreed with the household owner, the CB team placed the LT indoors and ITT outdoors, and then at the next household on the schedule the following day. The QA team followed this sequence but delayed by a day or two to enable them have at least two houses to re-survey that the CHW had surveyed no more than three days previously. The QA team conducted HLC indoors and outdoors in one of the two houses while the other was surveyed with LT indoors and ITT outdoors. During this process a QA team member slept in the ITT. On the following day, the pair of participants conducting HLCs would remain in the same house but would apply the LT and ITT methods, while the other pair also stayed in the same house as the previous night but applied HLC. Therefore, each cluster was visited for at least one night by the QA team with a lag of only a day or two after CB catches in or around the same houses. The only exception was cluster 10, which the QA team never visited because the households were closely situated to those of cluster 11. Therefore only one of the two clusters was sampled for convenience. A specific form was used to record the data including the cluster name, village name, household code, household owner name, date, and trapping method. All trapping methods were applied by the QA teams between 19:00 and 07:00 hours. The CHWs were informed in advance about the QA team so that they could conveniently get consent from the household owners for the additional days of mosquito collections.

For QA surveys, samples of mosquitoes were collected and morphologically differentiated to genus level individually in the field. Female *Anopheles* mosquitoes were further separated, recorded and preserved individually in microcentrifuge tubes over desiccated silica gel. All males were recorded and discarded.

### Mosquito processing in the laboratory

Further morphological identification of *Anopheles* to species group or complex [26] was conducted at the NMCC main laboratory. Female *Anopheles* samples were processed for detection of circumsporozoite protein ELISA [28], including confirmation following boiling of the head-thorax homogenates to prevent false-positive, [29] and polymerase chain reaction (PCR) identification of species within the *An. gambiae* complex [30] or *An. funestus* group [31].

### Entomological and epidemiological data analysis

Data were entered using Microsoft Excel 2007 and analysed using R statistical analysis software version 2.15.1, augmented with lattice, matrix and lme4 packages. To estimate the relative trapping efficiency of the different trapping schemes, a generalized linear mixed model (GLMM) was fitted using the number of mosquitoes of a given taxon as the Poisson-distributed dependent variable and trapping scheme as a categorical independent variable with five levels (CB-LT, CB-ITT, QA-HLC, QA-LT and QA-ITT). In order to account for spatial and temporal heterogeneity, as well as for over-dispersion, date, as well as households nested within clusters were treated as random effects. To ensure full comparability, data from the CB surveys collected more than seven days before or after a survey by the QA team in the same cluster were excluded from this analysis, so this comparison relates only to selected observations from the last three months of the study when both surveys were operational and overlapped in space and time.

Further, in estimating how *An. funestus* abundance predicts malaria infection risk among the human population, a GLMM was fitted with R statistical software augmented as above, with RDT results as the binomial dependent variable while the base 10 logarithm of the mean *An. funestus* catch per LT for each cluster, estimated from the Poisson model described above, was included as a continuous independent variable. Note, however, that to obtain specific estimates of the mean catches of *An. funestus* at each cluster, the model described above has to be modified so that cluster was treated as a categorical variable, rather than a random effect, and no intercept was included so that those estimates would be absolute rather than relative to an arbitrary reference group. Age categories of RDT-tested participants and date were treated as random effects and, to avoid any confounding effects household clustering would have on the *An. funestus* catch estimates, individual households were included nested within clusters as random effects. Data selected for this analysis of the dependence of malaria infection risk upon vector densities were restricted to the period from the onset of the study in January 2011 to September 2011 to avoid any confounding effects that the introduction of IRS in October and November 2011 would have on the densities or infection prevalence.

### Cost-effectiveness analysis

This QA exercise was conducted for only the three final months of the study (February to April, 2013) in 13 of the 14 clusters, each of which was visited at least once using motorized transport provided to the QA team for that period. The government employed technical team members and the driver received their normal *per diems* during this period, which were ZMW500 ($95.6) and ZMW300 ($57.4) per night, respectively. The cost incurred also included vehicle fuel, maintenance and depreciation (purchase cost of $15,000 depreciated to an expected value of $2,500 when disposed of by tender after five years of use) as well as the daily remuneration of the CHWs at the rate of ZMW100 ($19.1) per night of execution of the QA exercise. Whenever QA was conducted in clusters in Nyimba district, accommodation costs were also paid for the CHWs in the QA team because it was impractical for the team to return to their home in Chisobe village (Luangwa) on a daily basis, due to the long distance between the districts and the bad terrain between clusters during the rainy season. The CB CHWs received a minimal monthly incentive in form of the monthly remuneration of ZMW350 ($66.9) agreed upon at the start of the study. In addition to this incentive, the additional costs incurred included provision of field supplies and having their bicycles repaired in order to facilitate their ease of movement and carrying of the traps to the selected households where the trapping surveys took place.

In estimating these costs, the approximated amount of time and efforts spent on each trapping scheme was also factored into the total expenditure to calculate the cost per sampling night, as well as per single specimen of *An. funestus* collected. Consideration was restricted to *An. funestus* because it is overwhelmingly the most important vector mediating malaria transmission in this part of Zambia [21,32].

### Ethical considerations

This protocol was approved by the National Ethics Committee based at the University of Zambia (IRB00001131 of I0RG0000774) and the Ethical Review Board of the Liverpool School of Tropical Medicine (09.60). The benefits and risks associated with participating were explained to participants in advance of seeking their consent and assent. Adult participants had all consented to participate in the study, while all the children involved were permitted to do so by either their parents or guardians and provided assent where old enough. QA team members conducting HLC were administered with dapsone-pyrimethamine (Deltaprim^©^), one of the recommended drugs for chemoprophylaxis in Zambia, every week in accordance with the national guidelines.

## Results

### Species composition and abundances

A total of 20,683 female mosquitoes were collected by both the CB and QA sampling schemes in the 3,174 trap nights (Table 1). Morphological identification showed that the *An. funestus* group and *An. gambiae* complex comprised 34.5% (n = 7,127) and 3.3% (n = 685), respectively, while other anophelines and the culicines mosquitoes comprised 3.2% (n = 661) and 59.0% (n = 12,210), respectively, of the total. Of the 596 specimens that were initially identified as members of the *An. funestus* group by routine morphology, and then also successfully identified to species by PCR, 96.5% (n = 575) were confirmed to be *An. funestus sensu stricto*, with the remainder being *Anopheles rivulorum* (1.8%, n = 11) and *Anopheles leesoni* (1.7%, n = 10), respectively. Densities of the *An. funestus* group*,* as determined by routine morphological classification can therefore be considered quite a reliable representation of *An. funestus s.s.* as a species*.* PCR analysis of mosquitoes from the *An. gambiae* complex confirmed previous observations [21] that most specimens which amplified (69% (49/71)) were *An. quadriannulatus*, so this taxon is referred to as *An. quadriannulatus* subsequently in this text. All the other anophelines were morphologically identified as *Anopheles coustani* (34.0%; n = 225), *Anopheles pretoriensis* (22.5%; n = 149), *Anopheles rufipes* (19.1%; n = 126), *Anopheles squamosis* (13.2%; n = 87), *Anopheles implexus* (11.0%; n = 73) and one (0.2%) *Anopheles maculipalpis*.

**Table 1:** Total and unadjusted mean catches of malaria vectors and other mosquito species by community-based and quality assured sampling schemes

	**Quality assurance**				**Community-based**	
**Trapping method:**	**HLC indoor**	**HLC outdoor**	**LT**	**ITT**	**LT**	**ITT**
Person trap-nights	20	20	20	20	3171	2195
Number of houses sampled	20	20	20	20	505	432
Mean trap-nights per surveyed house	1.0	1.0	1.0	1.0	6.3	5.1
Mean trap-nights per cluster	1.5	1.5	1.5	1.5	226.6	156.8
Total catch of female mosquitoes						
*Anopheles funestus*	174	149	66	46	5,827	865
*Anopheles quadriannulatus*	10	2	0	0	613	60
Other anophelines	9	26	0	0	591	35
*Culex* species	426	394	94	82	9,548	1,666
Mean catch of female mosquitoes						
*Anopheles funestus*	8.7	7.5	3.3	2.3	1.8	0.4
*Anopheles quadriannulatus*	0.5	0.1	0.0	0.0	0.0	0.0
Other anophelines	0.5	1.3	0.0	0.0	0.2	0.0
Culex species	21.3	19.7	4.7	4.1	3.0	0.8

Of the total 550 *An. funestus s.l.* that were tested for circumsporozoite ELISA, only 23 *An. funestus* were detected with *Plasmodium falciparum* sporozoites in their salivary glands, corresponding to a sporozoite rate of 4.2%. This sporozoite infection prevalence is considerably higher than that previously reported from Chisobe [21], presumably because the period and geographical scope of sampling were far larger and also possibly because levels of insecticide resistance in the area may have increased. The abundance of *An. funestus s.s.* reported here across both districts is approximately consistent with previous studies at one of the clusters in Chisobe [21,32] and confirms that it is the predominant species sustaining malaria transmission in this part of Zambia.

### Sampling intensity and total catches of community-based trapping

**Figure 3:**
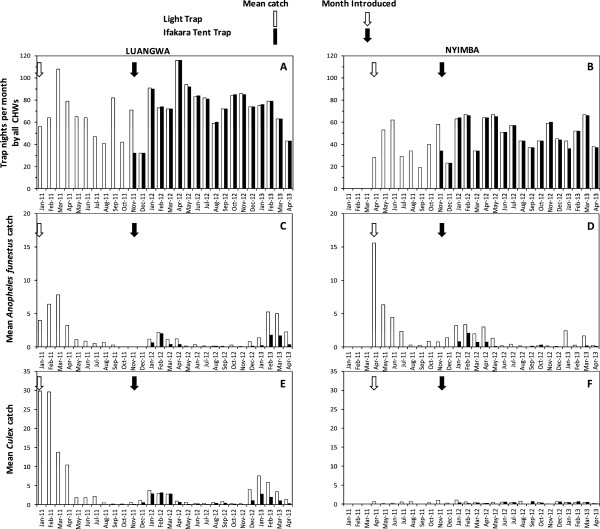
**Monthly trap-nights of community-based trapping schemes in the 14 clusters (A and B) and mean catches of *Anopheles funestus *** **(C and D) and *Culex *** **(E and F) in Luangwa and Nyimba districts.**

There was some inconsistency in the number of trap-nights of sampling by the CB trapping schemes over the 28 months of mosquito collections in all the clusters in both districts and the scheduled target sampling intensity was only occasionally achieved in Luangwa and never in Nyimba (Figure 3). It was only in February 2011 and April 2012 when trap-nights in Luangwa district exceeded the average of 150 trap-nights that had been expected to be attained per month per cluster. Nevertheless, adequate sampling to measure mean mosquito densities was sustained throughout the study. Interestingly, it appears that more trap-nights were conducted during the wet seasons when the CHWs observed increased abundance of *An. funestus* and *Culex* species (Figure 3). The overall numbers of person trap-nights conducted by the CB surveys were >100 greater than the QA surveys (Table 1), not only because the former had far greater numbers of staff operating, each of whom sampled with slightly greater frequency, but also because these were conducted over a much longer period of 28 months while the QA were restricted to the last three months of the study.

### Comparison of community-based and quality assurance mosquito trapping surveys

Summaries of the mean number of trap-nights of sampling per household and per cluster surveyed, mean catches and relative rates of capture for each taxon in times and places when both the CB and QA surveys were operational are shown in Table 2. The total numbers of person trap-nights and mean number of trap-nights completed per sampled cluster by the CB scheme were far higher (Table 2), despite the fact that inclusion of this data was restricted to within a week before or after a QA survey in the same cluster, simply because the frequency of sampling with a single, centralized QA team was limited by the practical logistical limitations described in background.

**Table 2:** Relative sampling sensitivity of community-based trapping scheme using CDC Light Traps and Ifakara Tent Traps to capture mosquitoes compared with quality assured catches when both operated simultaneously as estimated by generalized linear mixed models

	**Quality assurance**				**Community-based**	
**Trapping method**	**HLC indoor**	**HLC outdoor**	**LT**	**ITT**	**LT**	**ITT**
Person trap-nights	20	20	20	20	82	82
Number of houses sampled	20	20	20	20	76	76
Number of clusters surveyed	13	13	13	13	13	13
Mean trap-nights per surveyed house	1.0	1.0	1.0	1.0	1.1	1.1
Mean trap-nights per cluster	1.5	1.5	1.5	1.5	6.3	6.3
Total catch of female mosquitoes						
*Anopheles funestus*	174	149	66	46	126	24
*Anopheles quadriannulatus*	10	2	0	0	63	9
Other anophelines	9	26	0	0	36	1
*Culex* species	426	394	94	82	224	112
Mean catch [95% confidence interval]						
*Anopheles funestus*	4.507	3.860	1.498	1.047	0.584	0.101
	[2.115, 9.604]	[1.807, 8.244]	[0.680, 3.300]	[0.468, 2.343]	[0.284, 1.201]	[0.045, 0.227]
*Anopheles quadriannulatus*	0.097	0.019	0	0	0.184	0.026
	[0.025, 0.383]	[0.003, 0.127]	[NE]	[NE]	[0.086, 0.394]	[0.010, 0.070]
Other anophelines	0.005	0.014	0	0	0.016	0.000
	[0.001, 0.046]	[0.002, 0.124]	[NE]	[NE]	[0.004, 0.071]	[0.000, 0.005]
*Culex* species	11.941	11.044	1.743	1.374	0.305	0.146
	[5.186, 27.494]	[4.795, 25.439]	[0.771, 3.943]	[0.604, 3.126]	[0.145, 0.642]	[0.069, 0.312]
Relative rate of capture [95% confidence interval]						
*Anopheles funestus*	1.00	0.856	0.332***	0.232***	0.130***	0.022***
		[0.688, 1.065]	[0.185, 0.596]	[0.127, 0.426]	[0.079, 0.212]	[0.012, 0.041]
*Anopheles quadriannulatus*	1.00	0.200*	0	0	1.885	0.266
		[0.042, 0.959]	[NE]	[NE]	[0.497, 7.153]	[0.061, 1.157]
Other anophelines	1.00	2.889**	0	0	3.215	0.085
		[1.343, 6.213]	[NE]	[NE]	[0.355, 29.131]	[0.004, 1.740]
*Culex* species	1.00	0.925	0.146***	0.115***	0.026***	0.012***
		[0.807, 1.061]	[0.075, 0.283]	[0.059, 0.224]	[0.014, 0.047]	[0.007, 0.023]

*P < 0.05, **P < 0.01, ***P < 0.001.

For *An. funestus*, relative rate of capture per trap-night of the CB-LT was only 13% when compared with the indoor HLC, while that of CB-ITT was <3% (Table 2). However, comparing the CB-LT and the CB-ITT sampling methods with their application through the QA scheme, their relative capture rate per night of trapping was estimated to be 50% (relative rate (RR) [95% confidence interval (CI)] = 0.500 [0.299, 0.838]; P = 0.009) and 7% (RR [95% CI] = 0.069 [0.027, 0.174]; P < 0.001), respectively. Combined QA surveys with LT and ITT neither captured any *Anopheles quadriannulatus* nor any other anophelines in the three months these were conducted over. The CB-LT captured more *An. quadriannulatus* than any other method, including QA-HLC, but overall numbers of this mosquito were so low that this difference was not significant (Table 2)*.* Overall, CB trapping with either LT or ITT exhibited relatively low rates of capture compared with QA surveys of HLC and even with the same trapping methods when conducted simultaneously (Table 2).

Using the mean *An. funestus* trap catches (M_t_) by CB application of LT and ITT, as well as their relative capture rates compared with indoor HLC (λ_t_), as estimated by GLMM (Table 2) and the sporozoite prevalence estimate (S) described in the second paragraph of the results section, entomologic inoculation rates (EIR_t_) for each of the two traps of 68.6 and 70.1 infectious bites per unprotected per user were calculated (EIR_t_ = M_t_ × S × 365/λ_t_) assuming that the vast bulk of exposure of unprotected humans occurs indoors in this setting [32].

### Cost effectiveness of community-based and quality assurance surveys for capturing *Anopheles funestus*

Results for the QA-HLC placed indoors and outdoors were combined and considered as a single trapping method. Cost per sampling night was lowest for CB-LT, followed by CB-ITT, which was about twice as expensive, and then far more distantly by the QA survey, which were all at least an order of magnitude more expensive than either CB approach (Table 3). Cost per specimen of *An. funestus* captured was by far the lowest for CB-LT, followed by the potentially hazardous QA-HLC and then CB-ITT which were approximately five and seven times less cost effective, respectively, and then QA-LT and QA-ITT which were both an order of magnitude less cost effective than either CB method or QA-HLC (Table 3).

**Table 3:** **Crude estimates of the costs per sampling scheme per trap-night and per *Anopheles funestus *caught for the three months when community-based sampling was validated with quality assurance sampling schemes**

**Estimated parameter**	**Units**	**Quality assured**			**Community-based**	
		**QA-HLC**	**QA-LT**	**QA-ITT**	**CB-LT**	**CB-ITT**
Number of samples	Person-night	40	20	20	249	243
Numbers caught	Number of A*n. funestus*	526	41	32	637	156
Mean caught	Number of *An. funestus* per person-night	13.2	2.1	1.6	2.6	0.6
Personnel costs^a^	$(ZMW)	2,180(11,401.4)	1,520(7,949.6)	1,076(5,627.5)	2509.4(13,124.2)	2,939.4(15,373.1)
*Per diem* costs^b^	$(ZMW)	414(2,165.2)	1,243(6,500.9)	1,243(6,500.9)	621(3,247.8)	621(3,247.8)
Trap depreciation costs	$(ZMW)	0(0)	87.5(457.6)	125(653.8)	87.5(457.6)	125(653.8)
Transport costs^a^	$(ZMW)	225(1,176.8)	225(1,176.8)	225(1,176.8)	0(0)	0(0)
Vehicle maintenance costs^c^	$(ZMW)	212(1,108.8)	211(1,108.8)	212(1,108.8)	71(371.3)	71(371.3)
Vehicle depreciation cost^d^	$(ZMW)	2,500(13,075)	2,500(13,075)	2,500(13,075)	0(0)	0(0)
Bicycle repair costs^c^	$(ZMW)	0(0)	0(0)	0(0)	94(491.6)	611(3,195.5)
Bicycle depreciation costs^d^	$(ZMW)	0(0)	0(0)	0(0)	5(26.2)	5(26.2)
Total expenditure	$(ZMW)	5,531(28,927.1)	5,788(30,268.6)	5,381(28,142.6)	3,388(17,718.7)	4,372(22,867.7)
Cost per person-night of sampling	$(ZMW)	138.3(723.2)	289.4(1,513.4)	269.1(1,407.1)	13.6(71.2)	18.0(94.1)
Cost per specimen of *An. funestus* caught	$(ZMW)	10.5(55)	141.2(738.3)	168.2(879.5)	5.3(27.8)	28.0(146.6)

^a^Cost estimates were based on the approximated time and efforts spent on each trapping method.

^b^Assumptions made on the salaries paid and *per diem* to the central level teams during their visits.

^c^Estimated cost incurred for maintaining the equipment for transporting or visiting the trapping schemes per location.

^d^Monthly depreciation costs calculated when both trapping schemes where operational for three months.

$- US dollar.

*ZMW* - Zambian Kwacha.

Note: 1$ ≈ ZMW 5.23 which was the average exchange during the midpoint year of 2012.

### Epidemiological relevance of community-based surveys of *Anopheles funestus*

**Figure 4:**
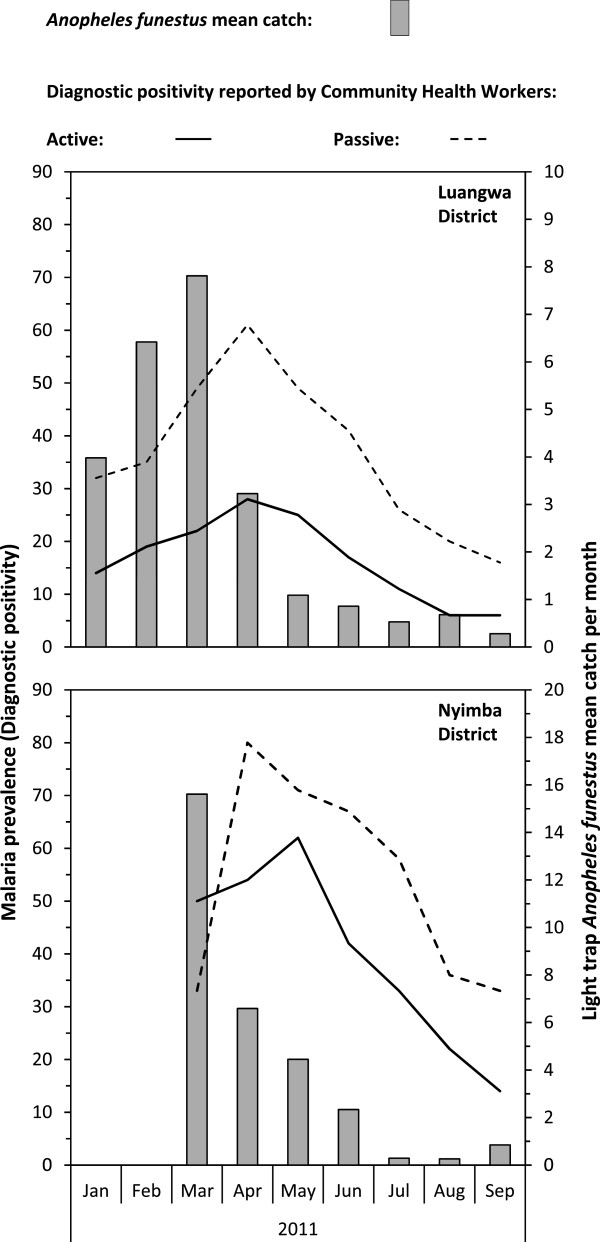
**Temporal variations of *Anopheles funestus *** **mean catches by light traps and the malaria diagnostic positivity among human residents from January to September 2011 in Luangwa and Nyimba districts.**

**Figure 5:**
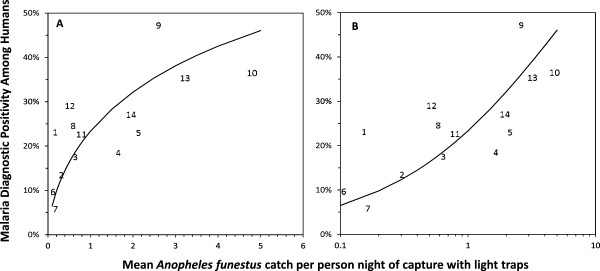
**Relationship between malaria diagnostic positivity among human residents and mean catches of *Anopheles funestus *per trap night of capture with light traps in each cluster, plotted with a standard (A) and logarithmic (B) horizontal axis.** Each data point represents the mean diagnostic positivity across all ages for a single cluster, numbered as described in Figure 2. The plotted lines represent the generalized linear mixed model fitted to these data, as described in the entomological and epidemiological data analysis section. Odds ratio [95% Confidence interval] for a ten-fold increase in the mean *Anopheles funestus* catch = 4.378 [2.438, 7.8580]; P < 0.001.

Figure 4 shows how the time-trends of malaria parasitaemia over the course of this period approximately follow those for the mean *An. funestus* catch by the CB entomological surveys. Consistent with previous studies in the area [33] parasite rates were generally much lower in Luangwa than in Nyimba district, with the least transmission recorded in the southernmost corner of the study area, at or near the district capital in Luangwa Boma, and these spatial trends in malaria parasitaemia were clearly associated with *An. funestus* density (Figure 5).

## Discussion

The CB trapping scheme proved to be far more practical, effective and cost effective for trapping large numbers of *An. funestus* because the higher frequency and overall numbers of mosquito samples collected within each population cluster captures temporal trends with far greater resolution and precision than conventional surveys by centralized teams, as exemplified by the QA surveys described herein. Familiarity of the CHWs with the communities and the collection sites enabled convenient, repeated, high frequency trapping in each cluster, simply because the CHWs live where they work. Overall, CB trapping with either LT or ITT exhibited relative low rates of capture per night of sampling compared with HLC, or even with the same trapping methods, implemented by the QA team. Nevertheless, CB trapping schemes caught far more mosquito of all taxa simply because these procedures allowed for more intensive sampling of each cluster in terms of trap-nights conducted over the whole period of the study. While longitudinal surveillance CB trapping scheme may not be as sensitive as the gold standard HLC in terms of estimating the absolute biting densities of host-seeking vectors, such assessments merely reflect the efficiency of the trapping method rather than the effectiveness of the system through which they are applied. When evaluated in terms of cost-effectiveness, it does appear to represent a far more affordable option for routine vector population dynamics monitoring at programmatic level that yields far more spatial and, especially, temporal resolution than is otherwise possible.

The only previous study to have validated the affordability, accuracy and epidemiological relevance of a CB trapping system relates to a municipal-scale platform for monitoring and evaluating the impact of an urban larviciding programme where *An. gambiae* s.s. is the predominant vector present [15]. The findings reported here also provide the first evidence of the applicability of quality assured CB trapping schemes in a transmissions system where local vectorial capacity is dominated by *An. funestus*. Unlike the preceding example from an urban Tanzanian setting, which necessarily relied on the locally designed and effective ITT [15], this study demonstrates for the first time how solar-recharged LT can be practically applied by CB staff to yield vector density data that predict malaria risk infection in 14 clusters distributed across >14,000 sq km of an isolated part of rural Zambia (Figures 4 and 5). The sampled clusters were far too widely distributed across these two districts for the QA team to visit more than once or twice every three months and these same logistical limitations are likely to apply to any centralized QA surveillance system with finite human and financial resources, especially if attempting to monitor vector populations on larger provincial or national scales. While others [34,35] have used CB trapping schemes to evaluate large-scale intervention progress, none conducted QA or estimated costs incurred under conditions comparable with programmatic operational conditions. The observations reported here therefore complement these earlier studies and provide another encouraging example of how much can be achieved by imparting basic entomological skills to non-specialist CB staff and availing them with minimal resources to monitor vector population dynamics and how it responds to control in their own communities.

Like all studies, this evaluation had limitations that merit careful consideration. The QA validation exercise was only carried out for three months during the rainy season so it can only be assumed, rather than proven, that these comparisons are representative of CHW and trap performance throughout the study. Furthermore, the CHWs were informed approximately one day in advance that the QA team would be coming to visit the cluster so it is possible that they conducted a small proportion of their trapping in that interim period more carefully than they normally would. Future studies of CB trapping schemes, especially those evaluating prototype systems operating at larger scales, should therefore incorporate continuous, randomized and unannounced, if not necessarily as intensive, QA surveys. It was also observed that the CHWs often conducted lower numbers of trap nights of sampling during the dry season when the catches were lowest because they thought it unnecessary to continue collecting even when the catches were often zero. It may, therefore, be necessary to sensitize CB staff to the critical importance of measuring the low but non-zero vector densities that occur in the dry season, especially in the context of any pre-elimination scenario where supplementary mass drug administration, mass screen and treat, or vector control measures are specifically introduced and evaluated as interventions to achieve termination of local transmission.

## Conclusions

Despite these study limitations, the prototype CB mosquito trapping scheme evaluated here clearly has considerable potential for improvement and scale-up. It is therefore recommended that future operational studies are undertaken to adapt, optimize and evaluate CB trapping schemes for monitoring mosquito population dynamics at nationally representative scales so that the influence of physiological and phenotypic traits, as determinants of success, limitations and failures of vector population control, can be assessed continuously, indefinitely and sustainably.

## Abbreviations

CB: Community-based; CHW: Community health worker; CS: Capsule suspension; ELISA: Enzyme-linked immunosorbent assay; ES: Emulsifiable suspension; GMEP: Global Malaria Eradication Programme; HLC: Human landing catch; IRS: Indoor residual spraying; NMCC: National Malaria Control Centre; NMCP: National Malaria Control Programme; IRB: Institutional Review Board; ITT: Ifakara Tent Trap; *kdr*: Knock down resistance; LT: Centers for Disease Control and Prevention miniature Light Traps; LLIN: Long-lasting insecticidal net; RDT: Rapid diagnostic test; QA: Quality assurance; ZMW: Zambian Kwacha.

## Competing interests

The authors declare that they have no competing interests.

## Authors’ contributions

GFK, AS, and CHS conceived and planned the study. CHS, AS, DC, JC, BH, and MK supervised execution of the study. NFL, MM and IM conducted the laboratory analyses. CHS analysed the data and drafted the manuscript in consultation with GFK. All authors contributed to editing of the manuscript. All authors read and approved the final manuscript.
